# Plant elicitor Peptides regulate root hair development in *Arabidopsis*

**DOI:** 10.3389/fpls.2024.1336129

**Published:** 2024-02-15

**Authors:** Yanping Jing, Fugeng Zhao, Ke Lai, Fei Sun, Chenjie Sun, Xingyue Zou, Min Xu, Aigen Fu, Rouhallah Sharifi, Jian Chen, Xiaojiang Zheng, Sheng Luan

**Affiliations:** ^1^ International Genome Center, Jiangsu University, Zhenjiang, China; ^2^ School of Life Sciences, Jiangsu University, Zhenjiang, China; ^3^ Chinese Education Ministry’s Key Laboratory of Western Resources and Modern Biotechnology, Key Laboratory of Biotechnology Shaanxi Province, College of Life Sciences, Northwest University, Xi’an, Shaanxi, China; ^4^ College of Life Sciences, Nanjing University, Nanjing, Jiangsu, China; ^5^ Department of Plant Protection, College of Agriculture and Natural Resources, Razi University, Kermanshah, Iran; ^6^ Department of Plant and Microbial Biology, University of California, Berkeley, Berkeley, CA, United States

**Keywords:** PROPEP, root hair growth, Ca signaling, regulatory mechanism, plant elicitor peptide (Pep)

## Abstract

Plant Elicitor Peptides (Peps) induce plant immune responses and inhibit root growth through their receptors PEPR1 and PEPR2, two receptor-like kinases. In our study, we found a previously unknown function of Peps that enhance root hair growth in a PEPRs-independent manner. When we characterized the expression patterns of *PROPEP* genes, we found several gene promoters of *PROPEP* gene family were particularly active in root hairs. Furthermore, we observed that *PROPEP2* is vital for root hair development, as disruption of *PROPEP2* gene led to a significant reduction in root hair density and length. We also discovered that *PROPEP2* regulates root hair formation via the modulation of *CPC* and *GL2* expression, thereby influencing the cell-fate determination of root hairs. Additionally, calcium signaling appeared to be involved in PROPEP2/Pep2-induced root hair growth. These findings shed light on the function of Peps in root hair development.

## 1 Introduction

Plants have developed highly conserved innate immune systems to protect themselves from external pathogens. These pathogens contain specific molecular patterns called PAMPs that are recognized by cell surface receptors in plants and result in PTI (pattern-triggered immunity) (Boller and Felix, 2009; Macho and Zipfel, 2014). Additionally, plants have the capability to release specific molecules termed damage- or danger-associated molecular patterns (DAMPs) in response to pathogen attacks or injuries, and these molecules also play a regulatory role in plant immunity (Endo et al., 2014; Macho and Zipfel, 2014). In Arabidopsis, a well-documented example of DAMPs is the family of plant elicitor peptides (Peps), which originate from the C-terminal regions of precursor proteins known as PROPEPs (Huffaker et al., 2006; Huffaker et al., 2013). Arabidopsis genome harbors eight *PROPEP*s, and they are responsible for generating eight small Pep peptides in response to pathogen invasion and physical injury (Huffaker et al., 2006; Bartels et al., 2013; Huffaker et al., 2013; Bartels and Boller, 2015; Klauser et al., 2015). Peps are recognized by a pair of closely related receptors, PEPR1 and PEPR2, which subsequently initiate downstream signaling events (Yamaguchi et al., 2006; Yamaguchi et al., 2010). These events include the elevation of cytosolic Ca^2+^ levels, the generation of reactive oxygen species, the expression of defense-related genes, the formation of calluses, lignin deposition, and inhibition of root growth (Millet et al., 2010; Bartels et al., 2013; Beck et al., 2014; Ma et al., 2014; Jing et al., 2019; Jing et al., 2020; Jing et al., 2023).

Root hairs are specialized tubular structures that develop from root epidermal cells. The dynamic adjustments in root hair growth, length, density, and morphology have a significant impact on the root’s surface area that determines the efficiency of nutrient and water uptake by plants, interactions between plants and microorganisms, and the stability of plant anchorage (Grierson et al., 2014). In some plant species, such as rice, all epidermal cells can differentiate into root hairs in a random manner (Kim et al., 2006; Kim and Dolan, 2011; Tominaga-Wada et al., 2013). In other species, such as Arabidopsis, only specific short epidermal cells have the potential to become root hairs.

In the well-established model of root hair development in *Arabidopsis*, the fate of root hair cells is determined by the position of epidermal cells (Datta et al., 2011; Grierson et al., 2014). Epidermal cells located exclusively outside of two underlying cortical cells are designated to differentiate into root hairs. Conversely, those with only one underlying cortical cell become non-hair cells (Datta et al., 2011). This cell fate determination is regulated by multiple transcription factors (TFs). Notably, the *TRANSPARENT TESTA GLABRA* (*TTG*), *GLABRA3* (*GL3*), *ENHANCER OF GLABRA3* (*EGL3*), and *WEREWOLF* (*WER*) TFs are expressed in non-hair cells, forming the WER-GL3/EGL3-TTG complex (Galway et al., 1994; Di Cristina et al., 1996; Masucci et al., 1996; Bernhardt et al., 2003; Schiefelbein, 2003). This complex plays a positive role in regulating *GLABRA2* (*GL2*), a central TF responsible for inhibiting root hair formation in non-hair cells (Di Cristina et al., 1996; Masucci et al., 1996; Datta et al., 2011). In contrast, the *CAPRICE* (*CPC*) and *TRIPTYCHON* (*TRY*) TFs promote the formation of root hairs by suppressing *GL2* expression (Schiefelbein, 2003; Grierson et al., 2014).

In our endeavor to unravel the signaling pathway of Peps in plants, our previous work highlighted Pep1’s role in stimulating root hair development when externally applied (Jing et al., 2019). In the present investigation, we provide evidence that both exogenous Peps and the overexpression of endogenous *PROPEP*s consistently promote the growth of root hairs. *PROPEP2* emerges as a critical regulator of root development, as the disruption of *PROPEP2* results in a significant reduction in both root hair density and length. Furthermore, we delve into the mechanism by which *PROPEP2* influences the determination of root hair cell fate through the modulation of CPC and GL2 expression. Simultaneously, PROPEP2 orchestrates calcium oscillations within root hair cells, directing the course of root hair development. These findings unveil a novel signaling pathway initiated by Pep/PROPEPs governing *Arabidopsis* root hair development.

## 2 Results

### 2.1 Regulation of root hair development by plant elicitor peptides

**Figure 1:**
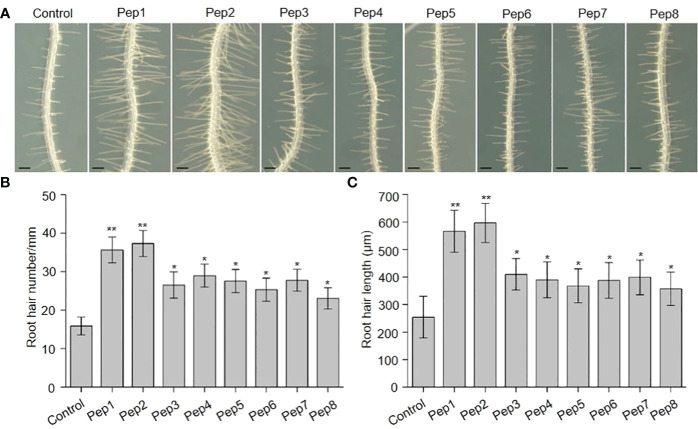
The effects of Peps on root hair development. **(A)** The growth phenotype of wild type root under Pep1-Pep8 treatment. Four-day-old WT plants were transplanted on half-strength Murashige and Skoog (MS) agar medium supplemented with or without 10 nM Pep1 to Pep8 for 48 h. Bars = 200 μm. **(B, C)** Statistics of the root hair number **(B)** and root hair length **(C)** as in **(A)**. Data are means ± SD (n = 15 roots per treatment). Asterisks in **(B, C)** indicate statistically significant differences compared with the untreated control. (Tukey’s test; *p < 0.05, **p < 0.01).

**Figure 2:**
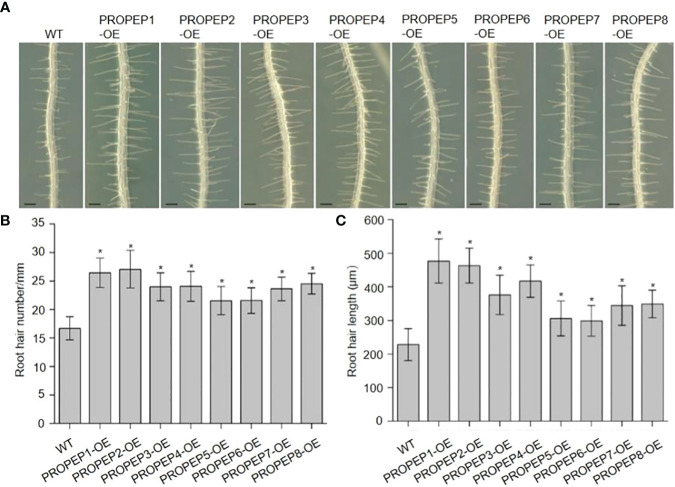
Over-expression of PROPEPs promote the root hair development. **(A)** The growth phenotype of root hair in wild type (WT)and wild type plants overexpressing *PROPEP*s (*PROPEP1-OE* to *PROPEP8-OE*). Four-day-old plants were transplanted on half-strength Murashige and Skoog (MS) agar medium for 48 h. Bars = 200 μm. **(B, C)** Statistics of the root hair number **(B)** and root hair length **(C)** as in **(A)**. Data are means ± SD (n = 15 roots per treatment). Asterisks in **(B, C)** indicate statistically significant differences compared with the WT plants. (Tukey’s test; *p < 0.05).

In previous investigations, we documented the immunomodulatory effects of Peps and their role in inhibiting root growth in *Arabidopsis* (Zheng et al., 2018; Jing et al., 2019; Shen et al., 2020). Additionally, seedlings treated with synthetic Pep1 or Pep2 caused intriguing root hair (RH)-related phenotypes. This led us to hypothesize that Peps might play a pivotal role in root hair development and growth. To scrutinize the impact of Peps on RH growth, we treated wild-type Col-0 (WT) seedlings with various synthetic Peps (Pep1-8) at 10 nM. Remarkably, all exogenous Peps significantly increased both the density and length of RH compared to controls (**Figure 1**). Notably, Pep1 and Pep2 nearly doubled both the number and length of RH (**Figure 1**). Furthermore, we engineered transgenic lines overexpressing each Pep precursor gene (*PROPEP*s) driven by the 35S promoter (**Supplementary Figures 1–8**). These overexpressed *PROPEP* lines exhibited higher RH density and longer RHs compared to the wild-type seedlings, mirroring the effects of exogenous Peps (**Figure 2**; **Supplementary Figures 1–8**). These results collectively suggested that both exogenous and endogenous Peps consistently promoted RH growth.

**Figure 3:**
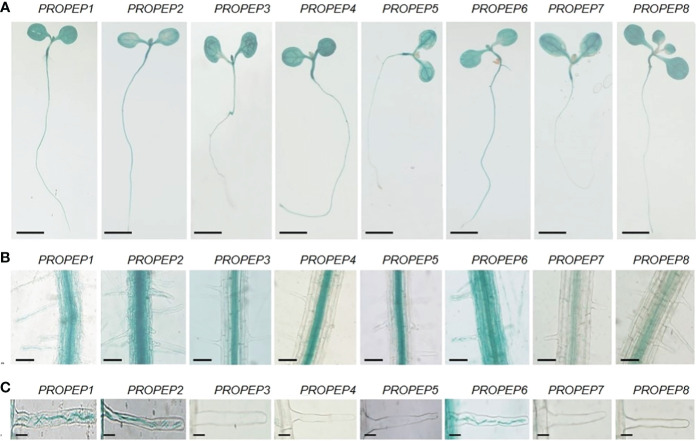
The tissue localization of PROPEPs. **(A)** Histochemical staining of GUS activity in 7-d-old transgenic plants harboring *proPROPEP1:GUS (PROPEP1)* to *proPROPEP8:GUS (PROPEP8)*. Bars =2 mm. **(B)** The GUS activity in roots of transgenic plants *PROPEP1* to *PROPEP8.* Bars =100 μm. **(C)** The GUS activity in root hairs of transgenic plants *PROPEP1* to *PROPEP8.* Bars =25 μm.

To elucidate the expression patterns of *PROPEP* genes to identify those naturally expressed in the RH, we generated transgenic lines with putative *PROPEP* promoters fused to a *β-glucuronidase* (GUS) gene reporter. GUS staining of these transgenic seedlings revealed extensive expression of all *PROPEP*s in both shoots and systems (**Figure 3A**). In root tissues, GUS activity in pro*PROPEP3/4/5/7/8:GUS* seedlings was primarily localized to vascular tissue, while the promoters of *PROPEP1/2/6* exhibited activity throughout the entire root (**Figure 3B**). Notably, the promoters of *PROPEP1/2/6* showed strong activity in root hair cells, implying their involvement in RH processes (**Figure 3C**). Additionally, we observed inducibility of *PROPEP* promoters by Peps, with GUS activity significantly enhanced in pro*PROPEP1/2/6:GUS* plants upon exposure to Peps (**Supplementary Figure 9**).

**Figure 4:**
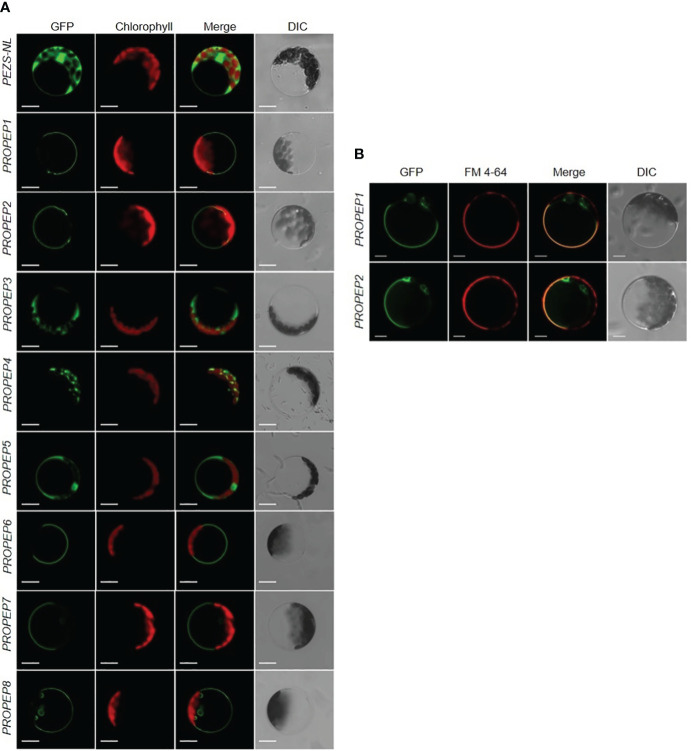
The subcellular localization assay of PROPEPs. **(A)** Arabidopsis mesophyll protoplasts were transiently transformed with a PEZS-NL vector expressed GFP signaling as the control. The coding sequence without the stop codon of *PROPEP1* to *PROPEP8* were cloned into pEZS-NL vector and transiently transformed into *Arabidopsis* mesophyll protoplasts. Columns from left to right show GFP signals (GFP), chlorophyll autofluorescence (Chlorophyll), merged images of GFP and chlorophyll (Merge), and bright-field differential interference contrast (DIC). Bars =5 μm. **(B)** FM 4-64 signaling was co-expressed with *PROPEP1-GFP* and PROPEP2-GFP in *Arabidopsis* protoplasts. The PROPEP1 and PROPEP2 fused GFP protein were transiently transformed into *Arabidopsis* mesophyll protoplasts and stained with 1 μM FM4-64 for 15 s before photographed. Columns from left to right show GFP signals (GFP), FM 4-64 fluorescence signals (FM 4-64), merged images of GFP and FM 4-64 (Merge), and bright-field differential interference contrast (DIC). Bars =5 μm.

To determine the subcellular localization of each Pep, we fused *PROPEP*s with the *Green Fluorescent Protein* gene (*PROPEP-GFP*) and transiently expressed them in *Arabidopsis* protoplasts. Despite the expected cytosolic localization based on function and the absence of a signal peptide, we unexpectedly observed cytosol-localized GFP signals exclusively in cells expressing *PROPEP3-GFP* or *PROPEP5-GFP* (**Figure 4A**). In contrast, *PROPEP1*, *PROPEP2*, *PROPEP6*, *PROPEP7*, and *PROPEP8* were found to target the tonoplast, while the GFP signal of *PROPEP4* overlapped with chloroplast fluorescence (**Figure 4A**). To validate the tonoplast localization of *PROPEP1* and *PROPEP2*, which have been extensively studied, we conducted lipophilic FM4-64 staining associated with plasma membrane. The GFP and FM4-64 fluorescence signals did not overlap (**Figure 4B**), confirming that GFP-PROPEP signals was not localized to the PM but the tonoplast.

### 2.2 Disruption of *PROPEP2* suppresses root hair development

**Figure 5:**
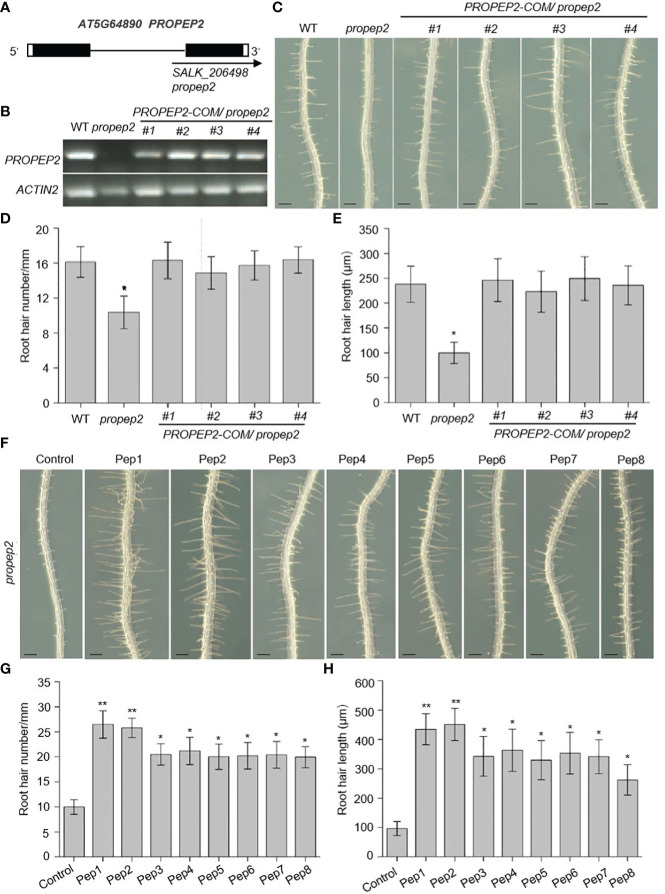
PROPEP2 regulates the root hair development. **(A)** Schematic map of T-DNA insertion location of *propep2* mutant. Black boxes, lines, and arrow represent exons, introns, and the position of the T-DNA insertion, respectively. The while boxes indicate the 5’or 3’UTRs. **(B)** RT-PCR analysis of the transcriptional level of *PROPEP2* in WT, *propep2* mutant and four independent complementation lines transformed with *PROPEP2* genomic DNA into *propep2* mutant (*PROPEP2-COM/propep2*). *Actin2* was used as internal standards. **(C)** The growth phenotype of root hairs in wild type (WT), *propep2* mutant and four *PROPEP2-COM/propep2* complementation lines. Four-day-old WT plants were transplanted on half-strength Murashige and Skoog (MS) agar medium for 48 h. Bars = 200 μm. **(D, E)** Statistics of the root hair number **(D)** and root hair length **(E)** as in **(C)**. Data are means ± SD (n = 15 roots per treatment). **(F)** The growth phenotype of root hairs in *propep2* mutant under Pep1-Pep8 treatment. Four-day-old plants were transplanted on half-strength Murashige and Skoog (MS) agar medium supplemented with or without 10 nM Pep1 to Pep8 for 48 h. Bars = 200 μm. **(G, H)** Statistics of the root hair number **(G)** and root hair length **(H)** as in **(F)**. Data are means ± SD (n = 15 roots per treatment). Asterisks in **(D, E, G, H)** indicate statistically significant differences compared with the control. (Tukey’s test; *p < 0.05, **p < 0.01).

To further investigate the role of the PROPEP family in root hair growth, we isolated transfer DNA (T-DNA) insertional mutants for each gene, aiming to assess their RH phenotypes. Unexpectedly, all of mutants failed to yield detectable T-DNA insertions, except for *propep2*. Notably, *propep2* (SALK_206498) contained a T-DNA insertion within the intron of *PROPEP2*. RT-PCR analyses demonstrated the absence of a full-length *PROPEP2* transcript in *propep2* (**Figures 5A, B**), confirming its status as a knockout mutant. Consequently, the *propep2* mutant exhibited a significant reduction in both RH density and length compared to wild-type (WT) seedlings (**Figures 5C–E**).

To corroborate that the observed RH phenotype in the *propep2* mutant was indeed caused by the T-DNA insertion, we generated complementation lines by introducing a genomic fragment of *PROPEP2* into the mutant. Remarkably, transgenic expression of *PROPEP2* in the mutant led to the restoration of *PROPEP2* transcript levels to a comparable level as in WT plants in four independent *PROPEP2-COM* lines (**Figure 5B**), fully rescuing the RH defect (**Figures 5C–E**). The Pep2 peptide was released from its precursor protein PROPEP2, the disruption of PROPEP2 could not synthesis the Pep2 peptide anymore, we further used the exogenous Pep2 peptide to analyzed the root hair formation in *propep2* mutant. As shown in **Figures 5F–H** and **Supplementary Figure 10**, supplementing the *propep2* mutant with synthesized Pep2 also resulted in the recovery of RH growth (**Figures 5F–H**; **Supplementary Figure 10**). These findings provide compelling evidence that *PROPEP2* plays an indispensable role in RH growth in *Arabidopsis*. Moreover, other Peps, in addition to Pep2, also restored RH growth in the *propep2* mutant, suggesting that other PROPEPs (such as PROPEP1 and PROPEP6) with expression in RH may also regulate RH growth (**Figures 5F–H**).

### 2.3 *PROPEP2* relies on *CPC* and *GL2* in regulating root hair formation

**Figure 6:**
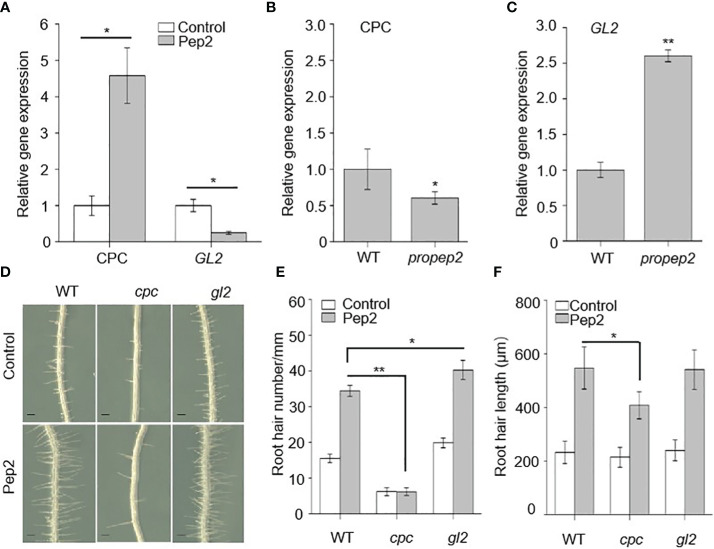
CPC and GL2 mediates the PROPEPs signals in root hair formation. **(A)** qRT-PCR analysis of *CPC* and *GL2* mRNA levels in wild type (WT) roots treated with 10 nM Pep2 for 24 h. The expression level of the untreated control (0 h) was set to 1.0, and Pep2 treatment levels were normalized to the control level. Data are means ± SD (n =3 individual reactions). **(B, C)** qRT-PCR analysis of *CPC* **(B)** and *GL2* **(C)** mRNA levels in 6-d-old WT and *propep2* roots. The expression level in WT root was set to 1.0. Data are means ± SD (n =3 individual reactions). **(D)** The growth phenotype of root hairs in wild type (WT), *cpc* and *gl2* mutant. Four-day-old plants were transplanted on half-strength Murashige and Skoog (MS) agar medium supplemented with or without 10 nm Pep2 for 48h. Bars = 200 μm. **(E, F)** Statistics of the root hair number **(E)** and root hair length **(F)** as in **(D)**. Data are means ± SD (n = 15 roots per treatment). Asterisks in **(A–C, E, F)** indicate statistically significant differences compared with the control. (Tukey’s test; *p < 0.05, **p < 0.01).

As Peps affect both the density and length of root hairs, we aimed to investigate whether PROPEPs/Peps work together with other components known to have a role in determining RH cell fate. We focused on examining the expression patterns of *GL2* and *CPC* in relation to Pep2 treatment. The *GL2* gene encodes a homeodomain-leucine zipper protein primarily expressed in non-hair cells, suppressing hair cell differentiation (Di Cristina et al., 1996; Masucci et al., 1996). On the other hand, *CPC* encodes a small protein containing a MYB-like DNA-binding domain, lacking a transcription activation domain, and it acts as a negative transcription regulator of *GL2*, indirectly promoting hair cell differentiation (Wada et al., 1997, 2002; Schellmann et al., 2002; Kirik et al., 2004). Initially, we assessed whether Pep2 impacts the expression levels of *CPC* and *GL2* using real-time RT-PCR. Following Pep2 treatment, *CPC* transcripts increased significantly, while *GL2* expression sharply decreased (**Figure 6A**). We next examined whether the expression pattern of *CPC* and *GL2* was altered in the *propep2* mutant. Interestingly, *CPC* mRNA levels decreased, whereas *GL2* expression increased significantly, opposite to the data from Pep2 treated samples (**Figures 6B, C**), suggesting that *PROPEP2* modulates RH growth, at least in part, by regulating *CPC* and *GL2* expression levels. Furthermore, all *PROPEP*-overexpressing (OE) lines showed increased *CPC* expression and decreased *GL2* mRNA levels (**Supplementary Figures 11A, B**), further supporting the notion that *PROPEP*s regulate the *CPC* and *GL2* expression.

We also examined mutant plants for *cpc* and *gl2*. Consistent with previous research, *cpc* mutant seedlings had sparse root hairs, while *gl2* mutants had more root hairs than WT plants (Masucci et al., 1996; Wada et al., 2002). The expression of *PROPEP2* in *cpc* and *gl2* mutant did not show significant differences compared with this in WT root (**Supplementary Figure 11C**). After Pep2 treatment, the increase in root hair density induced by Pep2 was compromised in *cpc* mutants, although Pep2-triggered root hair elongation persisted (**Figures 6D–F**), suggesting that *CPC* acts downstream of Pep2 signal to regulates the root hair formation. However, the root hair density in *gl2* mutant was further increased after Pep2 treatment, which displays significant difference compared with this in WT root (**Figures 6D–F**).

### 2.4 Calcium signaling may be involved in *PROPEP2*-mediated root hair growth

**Figure 7:**
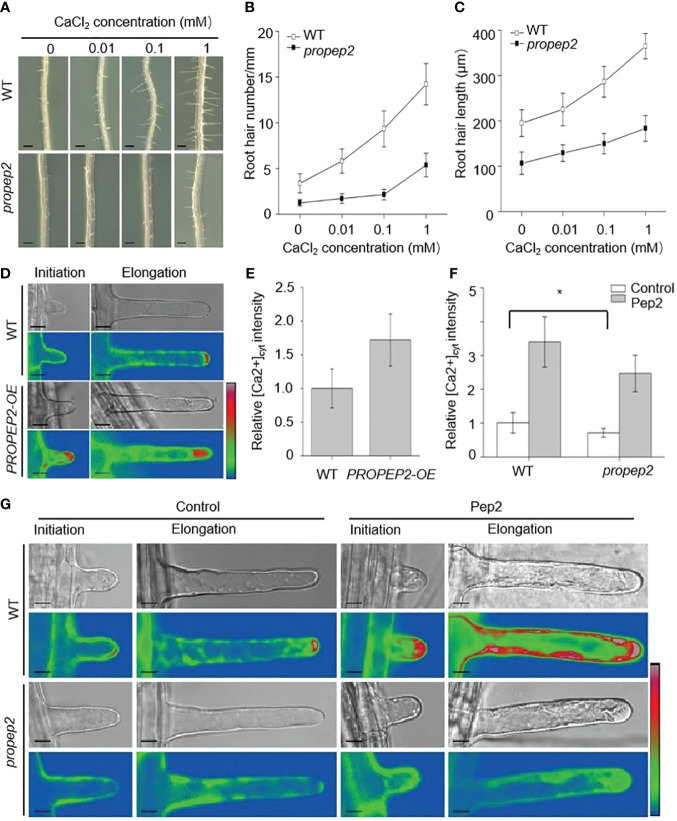
PROPEP2 mediates the root hair development dependent on Ca^2+^ concentrations changes. **(A)** The growth phenotype of root hairs in wild type (WT) and *propep2* mutant under CaCl_2_ treatment. Four-day-old plants were transplanted on half-strength Murashige and Skoog (MS) agar medium supplemented with or without 0.01, 0.1 and 1mM CaCl2 for 48 h. Bars = 200 μm. **(B, C)** Statistics of the root hair number **(B)** and root hair length **(C)** as in **(A)**. Data are means ± SD (n = 15 roots per treatment). **(D)** Imaging of Ca^2+^ fluorescence signals in the initiation and elongation root hairs. The 6-day-old wild type (WT) and *PROPEP2*-overexpression line (*PROPEP2-OE*) expressing the genetically encoded intracellular Ca^2+^ indicator GCaMP6s were used. Bars = 10 μm. **(E)** Quantitative analysis of cytosolic Ca^2+^ signals in the elongation root hair as in **(D)**, Relative fluorescence was normalized against that in WT root hairs (1.0). Data are mean ± SD; (n = 35 root hairs of 10 roots per treatment). **(F)** Quantitative analysis of cytosolic Ca^2+^ signals in the elongation root hairs of WT and *propep2* plants, 6-day-old plants were transplanted on half-strength Murashige and Skoog (MS) agar medium supplemented with or without 10 nm Pep2 for 6 h. Relative fluorescence was normalized against that in WT root hairs without Pep2 treatment (1.0). Data are mean ± SD; (n = 35 root hairs of 10 roots per treatment). **(G)** Imaging of Ca^2+^ fluorescence signals in the initiation and elongation root hairs of WT and *propep2* plants. Six-day-old WT and *propep2* plants expressing GCaMP6s were transplanted on half-strength Murashige and Skoog (MS) agar medium supplemented with or without 10 nm Pep2 for 6 h. Bars = 10 μm. A pseudocolor scale bar for relative cytosolic Ca^2+^ level calibration in **(D, G)** is shown on the right. Asterisks in **(F)** indicate statistically significant differences compared with the untreated control. (Tukey’s test; *p < 0.05).

Root hair growth is a finely tuned process in plants, regulated by a multitude of factors such as reactive oxygen species (ROS), cytoskeletal dynamics, and calcium signaling (Dunand et al., 2007; Pei et al., 2012; Zhang et al., 2016; Tan et al., 2019). Among these factors, the role of calcium, especially at the root hair tip, is critical for the elongation of these tubular cells (Takeda et al., 2008; Tan et al., 2019). To examine the link between Pep2 action and Ca signaling, we first observed that a reduction in calcium levels within the growth medium had an inhibitory effect on both the initiation and elongation of root hairs in wild-type (WT) seedlings (**Figures 7A–C**). Strikingly, the *propep2* mutant exhibited a complete absence of root hairs under conditions of reduced calcium availability (**Figures 7A–C**). Subsequently, we introduced the calcium-specific chelator EGTA into the growth medium. As the EGTA concentration increased, both root hair density and length in WT roots progressively decreased, ultimately leading to a complete absence of root hairs when EGTA concentrations reached 500 mM (**Supplementary Figure 12**). Concurrently, the effectiveness of Pep2 in promoting root hair growth diminished with the introduction of EGTA. Similarly, the *propep2* mutant encountered significant challenges in root hair development when exposed to EGTA concentrations exceeding 50 mM. However, the supplementation of Pep2 partially reinstated root hair growth in the mutant, although to a lesser extent than observed in WT seedlings (**Supplementary Figure 12**). These findings implied the pivotal role of external calcium availability in facilitating Pep2-induced root hair growth.

To delve deeper into the calcium dynamics occurring within root hairs, we introduced a fluorescent protein-based [Ca^2+^] _cytosol_ sensor, GCaMP6 (Gao et al., 2023). The GCaMP6 was expressed in WT plant under the control of the *UBQ10* promoter (Gao et al., 2023). To generate *PROPEP2-OE*/GCaMP6 plants, we utilized the homozygous pUBQ10:GCaMP6/WT plant as the background and introduced the PROPEP2-OE construct. To generate pUBQ10:GCaMP6/propep2 plants, we utilized the homozygous pUBQ10:GCaMP6/WT plant as the background and hybridized the pUBQ10:GCaMP6/WT with *propep2* mutant. As a result, the expression levels of GCaMP6 in various plant lines should be comparable. In *PROPEP2-OE* seedlings, the root hair tips exhibited significantly enhanced [Ca^2+^] _cytosol_ signals when compared to WT plants, both during the initiation and elongation stages of root hair growth (**Figures 7D–F**). Additionally, we generated *35S:PROPEP2-mRFP* transgenic plants, revealing extensive presence of PROPEP2 proteins in the root, including root hairs (**Supplementary Figure 13**). The [Ca^2+^] _cytosol_ signals in these plants were notably stronger than in WT, and these signals closely overlapped with PROPEP2-mRFP signals, emphasizing the correlation between *PROPEP2* expression and elevated [Ca^2+^] _cytosol_ levels (**Supplementary Figure 13**). Similarly, the application of Pep2 induced a substantial increase in [Ca^2+^] _cytosol_ levels, not only at the root hair tip but also throughout the entire root hair cell (**Figure 7G**; **Supplementary Figure 14**). These calcium dynamics were also observed with other Peps, mirroring the response to Pep2 (**Supplementary Figure 14**). In stark contrast, in the *propep2* mutant, there was an absence of discernible Ca^2+^ accumulation at the root hair tip (**Figure 7G**; **Supplementary Figure 15**). Moreover, the *PROPEP2* mutation resulted in a reduced response of Ca^2+^ elevation in root hairs to Pep2 compared to that in WT plants (**Figure 7G**; **Supplementary Figure 15**). We speculate that Pep2 intersects with Ca signaling to regulate root hair growth.

## 3 Discussion

Previous research has primarily focused on the immunomodulatory effects of Peps and their role in inhibiting overall root growth and have contributed significantly to our understanding of the functions of Peps in plant defense mechanisms. In this study, we demonstrate the multifaceted effects of Peps on root hair growth, unveil the expression patterns and subcellular localization of PROPEPs, and highlight the pivotal role of *PROPEP2* in this process. Additionally, we reveal the involvement of the CPC-GL2 module and calcium signature as downstream targets of PROPEPs/Peps in root hair differentiation, initiation, and elongation.

The development of plant root hairs is intricately regulated by a range of phytohormones, including auxins, ethylene, abscisic acid, and jasmonic acid. Phytohormones, notably auxins, primarily exert their influence on root hair development by promoting key processes such as root hair initiation, tip elongation, and the elongation of fully developed root hairs (Bruex et al., 2012; Lee and Cho, 2013; Zhang et al., 2016; Rymen et al., 2017). In addition to the pivotal role played by plant hormones in governing root hair development, recent scientific investigations have unveiled the significant involvement of hormone-like substances, specifically small peptides, in regulating various aspects of root hair development (Takahashi et al., 2019; Hsiao and Yamada, 2021). For instance, within plant cells, a class of small peptides known as Rapid Alkalinization Factors (RALFs) can be recognized by receptor-like kinases located in the plant cell membrane, such as FERONIA (FER) (Haruta et al., 2014). This recognition event triggers the formation of a protein complex involving FER and an intracellular receptor-like kinase called RIPK (Du et al., 2016). Together, they orchestrate the regulation of cytoplasmic alkalinization in root epidermal cells, consequently impacting the initiation of root hairs (Du et al., 2016). Another peptide, namely CLV3/ESR-related peptide 14 (CLE14), has been observed to enhance the expression of *CPC*, leading to the suppression of *GL2* transcription levels. This, in turn, promotes cell differentiation into hair cells, ultimately driving root hair development (Hayashi et al., 2018). In our study, we observed distinct tissue-specific expression patterns of PROPEPs, and their proteins exhibited varying subcellular localization. Notably, the introduction of exogenous synthetic Peps (Pep1-8) or the utilization of transgenic PROPEPs-OE lines led to a significant enhancement in both root hair density and length in seedlings. These results imply that Pep/PROPEPs play a pivotal role throughout all stages of root hair development, and the subcellular localization of Pep may not be directly correlated with its function in regulating root hair growth. Notably, *propep2* mutant seedlings with impaired root hair growth displayed elevated *GL2* levels but reduced *CPC* expression compared to WT seedlings. In contrast, both the transgenic *PROPEP-OE* lines and seedlings treated with Pep2 exhibited higher *CPC* expression but lower *GL2* levels. These observations imply that, akin to CLE14 (Hayashi et al., 2018), Pep/PROPEPs promote the differentiation of root hair cells by modulating the CPC-GL2 regulatory module.

The polarization and growth of cells, such as pollen tubes and root hairs, have been demonstrated to coincide with highly organized and polarized cytoplasmic contents (Rosen et al., 1964; Emons, 1987). Calcium, among other factors, plays a crucial role in activating proteins and enzymes that contribute to the organization of cytoskeletal elements and membrane structures necessary for the development and maintenance of cell polarity (Bush, 1995). A localized gradient of cytoplasmic free Ca^2+^ toward the growing apex has been observed in growing root hairs and pollen tubes, and the intensity of this gradient correlates with the growth rate of these cells (Pierson et al., 1996; Felle and Hepler, 1997; Wymer et al., 1997). In line with this, the deprivation of calcium in the medium resulted in the inhibition of root hair growth in both *propep2* and WT plants. Conversely, either overexpression of *PROPEP2* or supplementation of Pep2 significantly enhanced the tip calcium gradient of root hairs (**Figure 7**). Exploring the calcium channels/transporters or calcium signature elements expressed in root hairs would be intriguing for further elucidating the crosstalk between calcium oscillation and Peps-triggered root hair growth.

Regarding the perception of Peps, it has been established that PEPR1 and PEPR2 serve as the principal receptors responsible for transmitting the Pep signal and triggering corresponding responses, albeit with varying affinities for different Peps. Notably, the mutation of PROPEP2 resulted in stunted root hair growth in plants. In contrast to the *propep2* mutant, neither *pepr1*, *pepr2*, nor the double mutant *pepr1 pepr2* displayed any discernible root hair deficit phenotype (**Supplementary Figure 16**). However, it is worth highlighting that exogenous application of Pep2 failed to stimulate root hair growth in *pepr1 pepr2*, underscoring the exclusive role of PEPR1/2 as the receptors for perceiving exogenous Peps (**Supplementary Figure 16**). Recent research introduced sucrose-induced receptor kinase 1 (SIRK1) as a novel receptor for Pep7, orchestrating sucrose-mediated water flux regulation and lateral root development (Wang et al., 2022). Consequently, we posit the existence of an unidentified perception system within the cell, which may facilitate the sensing of Peps and subsequently regulate root hair growth. In light of this, unraveling the signaling pathways downstream of Peps and PROPEPs becomes imperative for a holistic comprehension of root hair development. The identification of novel receptors and components participating in these pathways promises valuable insights into the mechanisms by which Peps govern root hair fate and growth.

## 4 Materials and methods

### 4.1 Plant materials and growth conditions

*Arabidopsis* (*Arabidopsis thaliana*) mutant lines *propep2* (SALK_206498), *cpc* (Wada et al., 2002), *gl2* (Wang et al., 2010) and transgenic line p*UBQ10:GCaMP6s* (Gao et al., 2023) were described previously. Homozygous mutant plants were identified by RT-PCR or DNA sequencing using primers described in **Supplementary Table 1**. The seedlings were grown on half-strength Murashige and Skoog (MS) medium, containing 1% sucrose and 0.8% phytogel (Sigma-Aldrich, St. Louis, MO, USA) in the growth chamber. The growth conditions had 90 μmol/m^2^/s light intensity with a 16 h light/8 h dark photoperiod at 22°C.

### 4.2 Peptide synthesis

The peptides used in this study were synthesized by GL Biochem. The sequences (from the N terminus to the C terminus) were as follows:

Pep1, ATKVKAKQRGKEKVSSGRPGQHN;

Pep2, DNKAKSKKRDKEKPSSGRPGQTNSVPNAAIQVYKED;

Pep3, EIKARGKNKTKPTPSSGKGGKHN;

Pep4, GLPGKKNVLKKSRESSGKPGGTNKKPF;

Pep5, SLNVMRKGIRKQPVSSGKRGGVNDYDM;

Pep6, ITAVLRRRPRPPPYSSGRPGQNN;

Pep7, VSGNVAARKGKQQTSSGKGGGTN;

Pep8, GGVIVKSKKAARELPSSGKPGRRN;

### 4.3 Plasmid constructions and plant transformation

To produce transgenic *proPROPEP1:GUS* to *proPROPEP8:GUS* lines, the promoter regions upstream of the start codons of *PROPEP1* (1640-bp), *PROPEP2* (765-bp), *PROPEP3* (1437-bp), *PROPEP4* (1197-bp), *PROPEP5* (798-bp), *PROPEP6* (1707-bp), *PROPEP7* (1170-bp), *PROPEP8* (678-bp) were amplified and cloned into the *p*CAMBIA1300-GUS binary vector. For the transgenic PROPEP1 to PROPEP8 over-expression lines (termed as PROPEP1-OE to PROPEP8-OE), the coding sequence (CDS) without the stop codon of *PROPEP1* to *PROPEP8* were cloned into pEZS-NL to generate 35S-PROPEPs-GFP constructs and then cloned into the pART27 binary vector. The constructs were transformed into *Agrobacterium tumefaciens* strain GV3101 and further transformed into wild type plants using the floral-dip method (Clough and Bent, 1998). For construction of the genetic PROPEP2-OE harboring *GCaMP6s*, the CDS without the stop codon of *PROPEP2* was cloned into pEZS-NL to fuse with mRFP. The 35S-PROPEPs-mRFP construct was cloned into pART27 binary vector and then transformed into *pUBQ10:GCaMP6s* plants through GV3101 infection of floral-dip.

For construction of the genetic *PROPEP2* complementary lines (termed as *PROPEP2-COM*/*propep2*), the full-length genomic DNA of the PROPEP2 fragment (a 1557-bp fragment containing a 590-bp promoter and a 967-bp genomic region from translation initiation codon to 3’ UTR domain) was amplified from the genomic DNA of wild-type seedlings and cloned into the binary vector pCAMBIA-1300. Then, the recombinant plasmid was transformed into the *Agrobacterium tumefaciens* strain GV3101 and further transformed into the propep2 mutant using the floral-dip method. The primers used to produce the constructs are listed in **Supplementary Table 1**.

### 4.4 Root hairs growth analyze

The root hairs growth was analyzed as previously described (Tan et al., 2019) with modifications. In brief, 4-day-old seedlings were transferred onto half-strength MS agar medium supplemented with different treatment conditions, and the plates were placed vertically in growth room for another 48h. The roots were covered by cover glass to push the angle of root hairs parallel to the surface of solid medium. Roots were photographed under an SZX16 microscope (Olympus). The 2 mm root hair distribution zone, which located 0.5 cm far away from the root tip was counted by using Image J software to analyze the root hairs length and root hair number. No less than 15 roots were analyzed for each treatment, three independent repetitions were performed.

### 4.5 RT-PCR and qRT-PCR analysis

Total RNA in roots were extracted using the TRIzol reagent (Invitrogen, Carlsbad, CA, USA), according to the manufacturer’s protocol. Two μg RNA was used to synthesis the cDNA by using M-MLV Reverse Transcriptase (Promega, Madison, WI, USA). Real time qRT-PCR analysis was performed using the FastStart Universal SYBR Green mastermix (Roche Diagnostics, Hong Kong) on a CFX Connect Real Time System (Bio-Rad, Berkeley, CA, USA) using *Actin2* as internal standards. All individual reactions were performed in triplicate. The primers used are listed in **Supplementary Table**.

### 4.6 Histochemical GUS analysis

GUS activity was detected by histochemical staining of tissues as previously described (Liu et al., 2015). Briefly, T2 transgenic seedlings were incubated in GUS staining solution (2 mM 5-bromo-4-chloro-3-indolyl-b-D-glucuronide, 1 mM K_3_Fe(CN)_6_, 1 mM K_4_Fe(CN)_6_·3H_2_O, 10 mM Na_2_EDTA, 0.1% Triton X-100, and 50 mM Na_3_PO_4_, pH 7.0) at 37°C for 6 h. After the tissue with 75% (vol/vol) ethanol was sufficiently decolorized to remove chlorophyll, individual representative plant tissues, the roots and root hairs were photographed under a microscope (Olympus, SZX16) equipped with a camera.

### 4.7 Subcellular localization assays in planta

Subcellular localization assays were performed as previously described (Mao et al., 2014) with slight modifications. Briefly, 4-week-old Arabidopsis rosette leaves were digested by Cellulase R-10 and Macerozyme R-10 (Yakult Pharmaceutical) to prepare the mesophyll protoplasts. The protoplasts were resuspended with suspension solution (154 mM NaCl, 125 mM CaCl_2_, 5 mM KCl, 2 mM 4-Morpholineethanesulfonic acid (MES) adjusted to pH 5.7 with KOH) and further transfected with 20 μg recombinant plasmid DNA (PROPEP1 to PROPEP8-pEZS-NL-GFP) by using polyethylene glycol-mediated transformation protocol (Sheen, 2001). The transformed protoplasts were incubated in the dark at 23°C for 16 h before confocal imaging analysis. Imaging was performed on an LSM-710 argon/krypton laser scanning confocal microscope (Zeiss) with a 63 × objective. FM 4-64 excitation at 514 nm and emission at 600-700 nm. GFP signals were excited at 488 nm wavelength and collected emission between 495 and 550 nm. Z-stack images were collected with 1 μm steps and the scan speed was 8 s/scan.

### 4.8 Root hairs calcium imaging

For Peps-induced root hairs [Ca^2+^]_cytosol_ signals assays, the 6-day-old seedlings expressing GCaMP6s were supplemented with or without Peps for 6 h, the root hairs harboring GCaMP6s were monitored by a LSM-710 confocal microscope with a 20 × objective. The interval of data acquisition was 10 seconds, the Z-stack images were acquired from top to bottom of the cells with 1 μm steps and the scan speed was 6 s/scan. The excitation wavelengths for [Ca^2+^]_cytosol_ fluorescence signals was 488 nm. To quantitatively analyze fluorescence intensity, confocal images were captured under strictly identical acquisition parameters, which included laser power, photomultiplier settings, offset, zoom factor, and resolution, across all experimental root samples. The fluorescence intensity was analyzed by Image J software.

### 4.9 Statistical analysis

For all experiments, three independent repetitions were performed. One way ANOVA Tukey’s test was used for statistical analysis. Asterisks in the figures denote significant differences as follows: *P < 0.05, **P < 0.01, and ***P < 0.001.

## Data availability statement

The original contributions presented in the study are included in the article/**Supplementary Material**. Further inquiries can be directed to the corresponding authors.

## Author contributions

YJ: Conceptualization, Data curation, Formal analysis, Funding acquisition, Investigation, Writing – original draft, Writing – review & editing. FZ: Data curation, Writing – original draft, Writing – review & editing. KL: Data curation, Writing – review & editing. FS: Data curation, Writing – review & editing. CS: Investigation, Writing – review & editing. XYZ: Investigation, Writing – review & editing. MX: Writing – original draft, Writing – review & editing. AF: Writing – original draft, Writing – review & editing. JC: Conceptualization, Formal analysis, Funding acquisition, Writing – original draft, Writing – review & editing. XJZ: Conceptualization, Data curation, Formal analysis, Funding acquisition, Writing – original draft, Writing – review & editing. SL: Conceptualization, Project administration, Resources, Supervision, Writing – original draft, Writing – review & editing. RS: Data curation, Writing – review & editing.
