# Supplementary material for: Plant elicitor Peptides regulate root hair development in Arabidopsis
Source: Front Plant Sci. 2024 Feb 15;15:1336129. doi: 10.3389/fpls.2024.1336129 (PMC10902123; doi:10.3389/fpls.2024.1336129)
Supplement: Supplementary file 1 [file DataSheet_1.docx]

**Supplemental figures and legend**

**
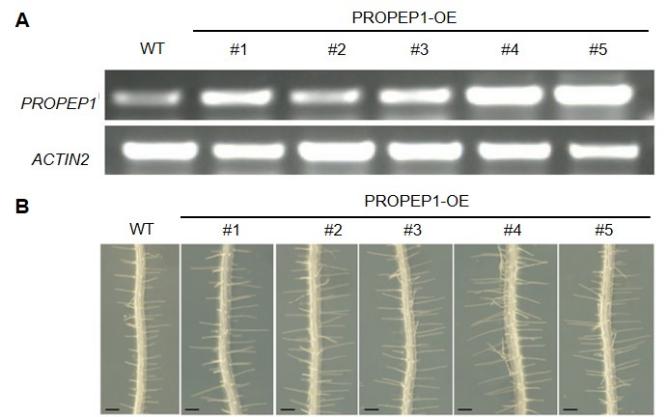
**

**Supplemental Figure 1. Identification of PROPEP1 over-expression lines.** (A)RT-PCR analysis of the transcriptional level of *PROPEP1* in WT and five *PROPEP1* overexpressing lines (*PROPEP1-OE*) transformed with PROPEP1 coding sequence without the stop codon into WT plant. *ACTIN2* was used as internal standards. (B) The growth phenotype of root hairs in WT and five *PROPEP1-OE* plants. Four-day-old seedlings were transplanted on half-strength Murashige and Skoog (MS) agar medium for 48 h. Bars = 200 μm.

**
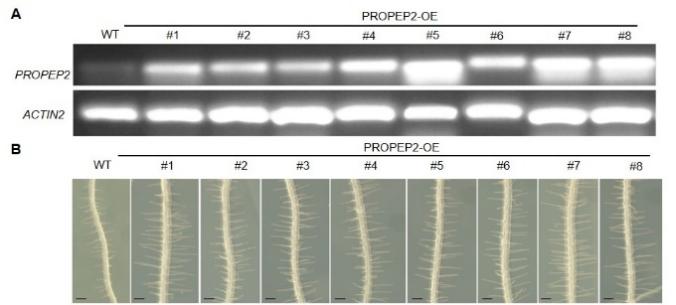
**

**Supplemental Figure 2. Identification of PROPEP2 over-expression lines.** (A)RT-PCR analysis of the transcriptional level of *PROPEP2* in WT and eight *PROPEP2* overexpressing lines (*PROPEP2-OE*) transformed with *PROPEP2* coding sequence without the stop codon into WT plant. *ACTIN2* was used as internal standards. (B) The growth phenotype of root hairs in WT and eight *PROPEP2-OE* plants. Four-day-old seedlings were transplanted on half-strength Murashige and Skoog (MS) agar medium for 48 h. Bars = 200 μm.

**
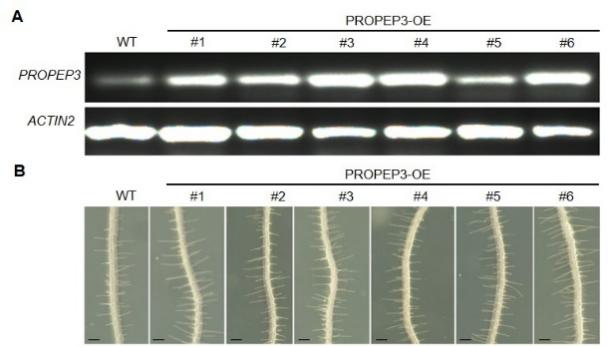
**

**Supplemental Figure 3. Identification of PROPEP3 over-expression line.** (A) RT-PCR analysis of the transcriptional level of *PROPEP3* in WT and six *PROPEP3* overexpressing lines (*PROPEP3-OE*) transformed with *PROPEP3* coding sequence without the stop codon into WT plant. *ACTIN2* was used as internal standards. (B) The growth phenotype of root hairs in WT and six *PROPEP3-OE* plants. Four-day-old seedlings were transplanted on half-strength Murashige and Skoog (MS) agar medium for 48 h. Bars = 200 μm.

**
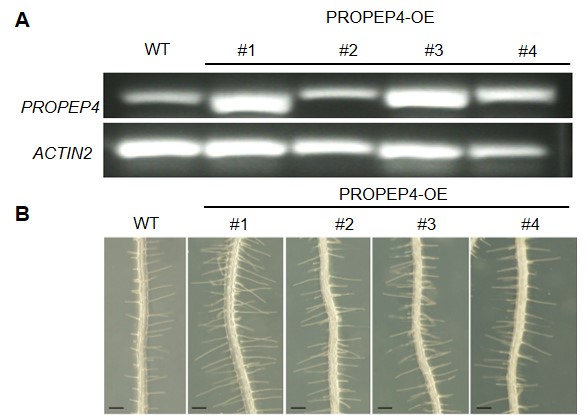
**

**Supplemental Figure 4. Identification of PROPEP4 over-expression lines.** (A) RT-PCR analysis of the transcriptional level of *PROPEP4* in WT and four *PROPEP4* overexpressing lines (*PROPEP4-OE*) transformed with *PROPEP4* coding sequence without the stop codon into WT plant. *ACTIN2* was used as internal standards. (B) The growth phenotype of root hairs in WT and four *PROPEP4-OE* plants. Four-day-old seedlings were transplanted on half-strength Murashige and Skoog (MS) agar medium for 48 h. Bars = 200 μm.

**
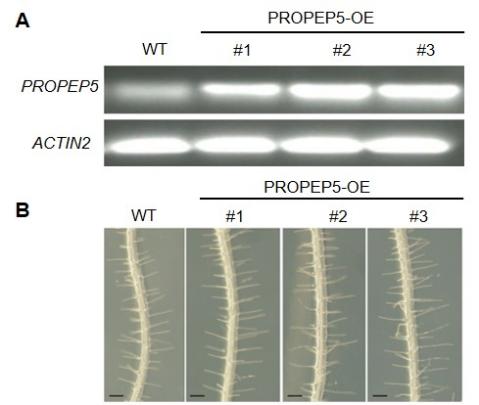
**

**Supplemental Figure 5. Identification of PROPEP5 over-expression lines.** (A) RT-PCR analysis of the transcriptional level of *PROPEP5* in WT and three *PROPEP5* overexpressing lines (*PROPEP5-OE*) transformed with *PROPEP5* coding sequence without the stop codon into WT plant. *ACTIN2* was used as internal standards. (B) The growth phenotype of root hairs in WT and three *PROPEP5-OE* plants. Four-day-old seedlings were transplanted on half-strength Murashige and Skoog (MS) agar medium for 48 h. Bars = 200 μm.

**
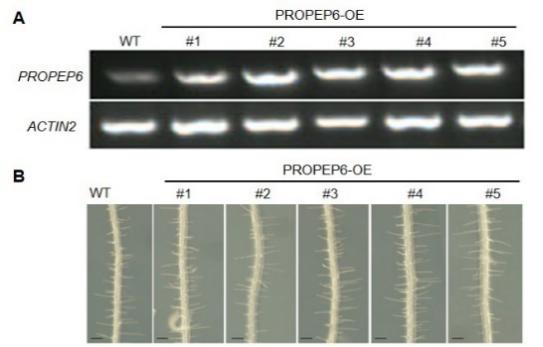
**

**Supplemental Figure 6. Identification of PROPEP6 over-expression lines.** (A) RT-PCR analysis of the transcriptional level of *PROPEP6* in WT and five *PROPEP6* overexpressing lines (*PROPEP6-OE*) transformed with *PROPEP6* coding sequence without the stop codon into WT plant. *ACTIN2* was used as internal standards. (B) The growth phenotype of root hairs in WT and five *PROPEP6-OE* plants. Four-day-old seedlings were transplanted on half-strength Murashige and Skoog (MS) agar medium for 48 h. Bars = 200 μm.

**
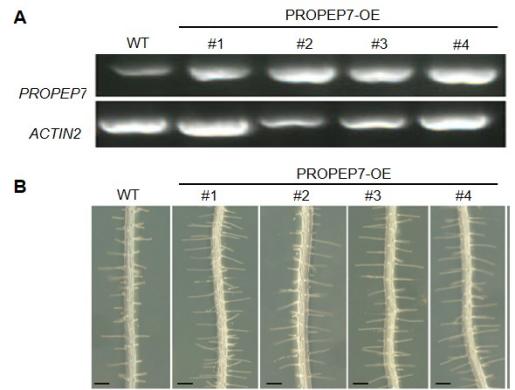
**

**Supplemental Figure 7. Identification of PROPEP7 over-expression lines.** (A) RT-PCR analysis of the transcriptional level of *PROPEP7* in WT and four *PROPEP7* overexpressing lines (*PROPEP7-OE*) transformed with *PROPEP7* coding sequence without the stop codon into WT plant. *ACTIN2* was used as internal standards. (B) The growth phenotype of root hairs in WT and four *PROPEP7-OE* plants. Four-day-old seedlings were transplanted on half-strength Murashige and Skoog (MS) agar medium for 48 h. Bars = 200 μm.

**
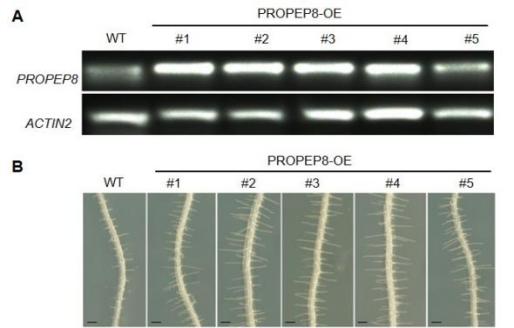
**

**Supplemental Figure 8. Identification of PROPEP8 over-expression lines.** (A) RT-PCR analysis of the transcriptional level of *PROPEP8* in WT and five *PROPEP8* overexpressing lines (*PROPEP8-OE*) transformed with *PROPEP8* coding sequence without the stop codon into WT plant. *ACTIN2* was used as internal standards. (B) The growth phenotype of root hairs in WT and five *PROPEP8-OE* plants. Four-day-old seedlings were transplanted on half-strength Murashige and Skoog (MS) agar medium for 48 h. Bars = 200 μm.

**
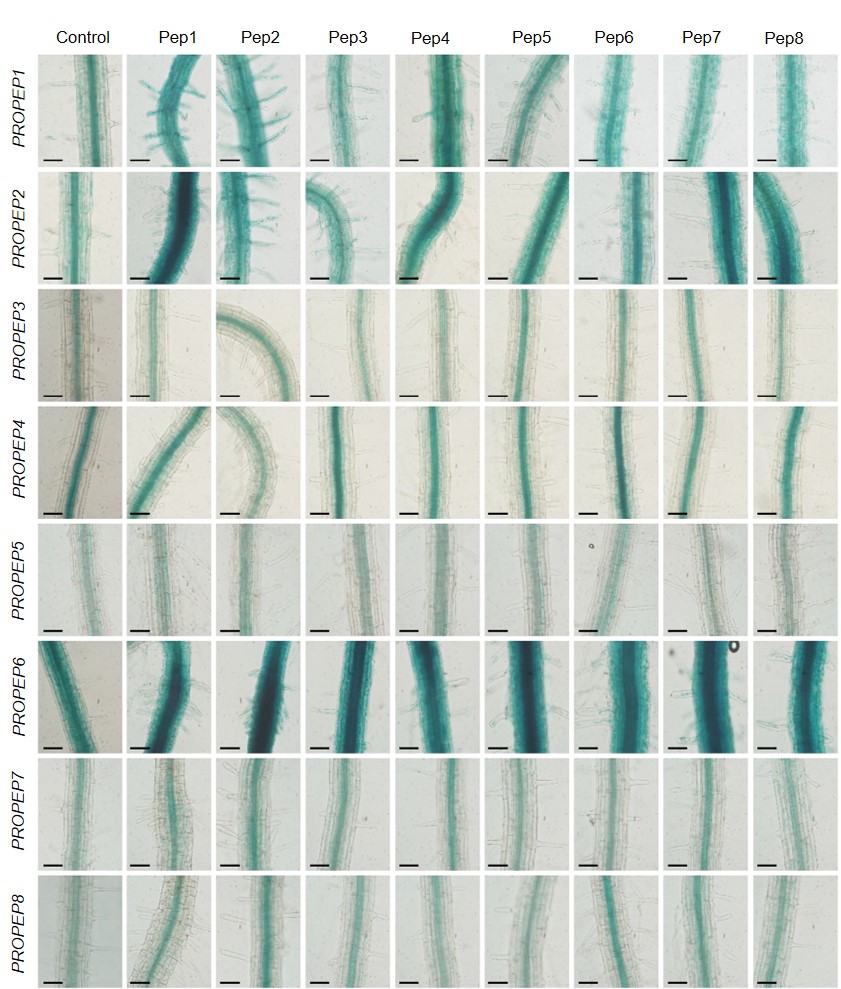
**

**Supplemental Figure 9. The effect of Peps on *PROPEP*s expression in root tissues.** Histochemical staining of GUS activity in roots of transgenic plants harboring *proPROPEP1:GUS (PROPEP1)* to *proPROPEP8:GUS (PROPEP8).* Six-day-old plants were transplanted on half-strength Murashige and Skoog (MS) agar medium supplemented with or without 10 nm Pep1 to Pep8 for 12 h. Bars =100 μm.

**
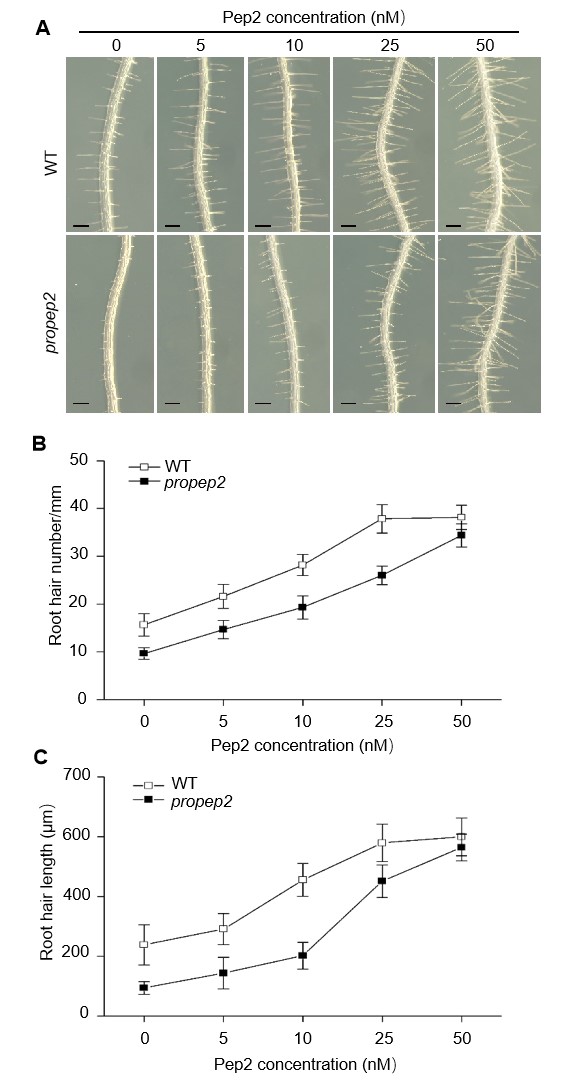
**

**Supplemental Figure 10. The effect of Pep2 on root hair development in WT and *propep2* mutants.** (A) The growth phenotype of root hairs in wild type (WT) and *propep2* mutant under Pep2 treatment. Four-day-old plants were transplanted on half-strength Murashige and Skoog (MS) agar medium supplemented with or without 0, 5, 10 and 25 and 50 nM Pep2 for 48 h. Bars = 200 μm. (B) and (C) Statistics of the root hair number (B) and root hair length (C) as in (A). Data are means ± SD (n = 15 roots per treatment).

**
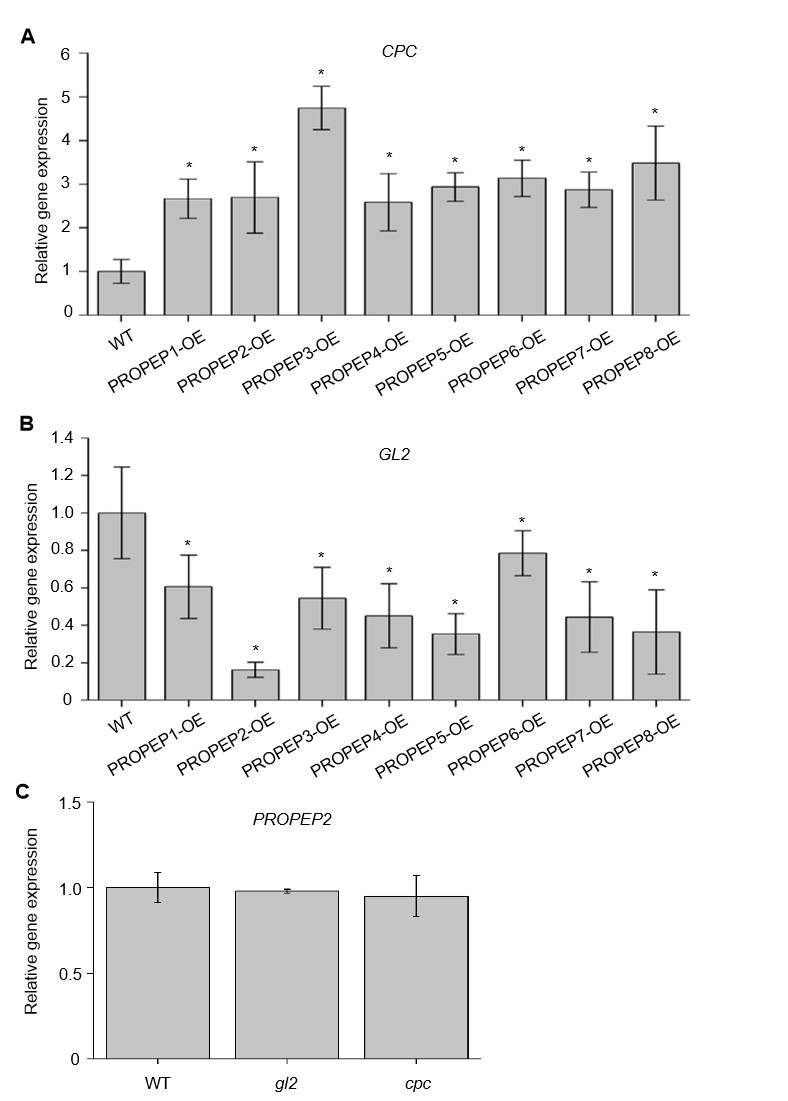
**

**Supplemental Figure 11. The expression of *CPC* and *GL2* in PROPEPs over expression lines.** (A) and (B) qRT-PCR analysis of *CPC* (A) and *GL2* (B) mRNA levels in root of wild type (WT) and *PROPEP*s over expression lines. (C) qRT-PCR analysis of PROPEP2 mRNA levels in root of wild type (WT), *gl2* and *cpc* mutant. Six-day-old plants were used. The expression level in WT root was set to 1.0. Data are means ± SD (n =3 individual reactions). Asterisks in (A) and (B) indicate statistically significant differences compared with the control. (Tukey's test; *p < 0.05).


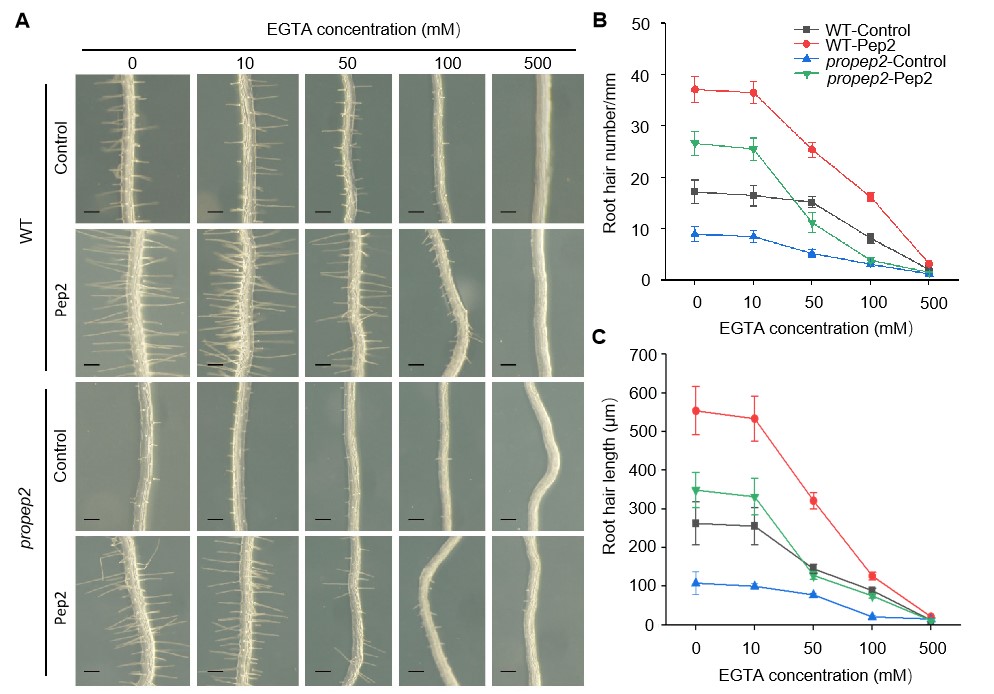


**Supplemental Figure 12. The effect of EGTA on root hair development.** (A)The growth phenotype of root hairs in wild type (WT) and *propep2* mutant. Four-day-old plants were transplanted on half-strength Murashige and Skoog (MS) agar medium supplemented with or without 25 Pep2 added with or without EDTA (ranged from 10 to 500 mM) for 48 h. Bars = 200 μm. (B) and (C) Statistics of the root hair number (B) and root hair length (C) as in (A). Data are means ± SD (n = 15 roots per treatment).


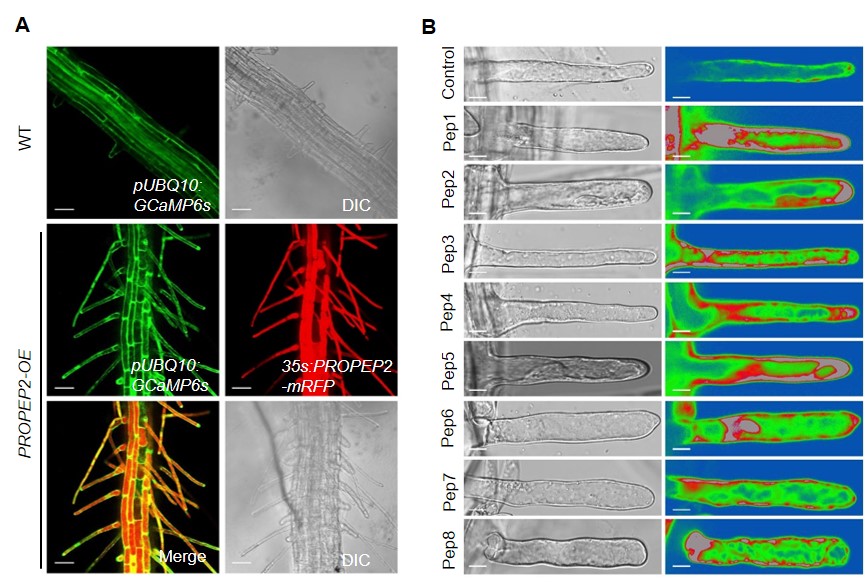


**Supplemental Figure 13. Ca^2+^ fluorescence signals in root.** The 6-day-old wild type (WT) and *PROPEP2*-overexpression line (the coding sequence without the stop codon of *PROPEP2* fused with *mRFP* to generate *35S-PROPEP2-mRFP* transgenic plants) expressing the genetically encoded intracellular Ca^2+^ indicator GCaMP6s. Bars = 100 μm.


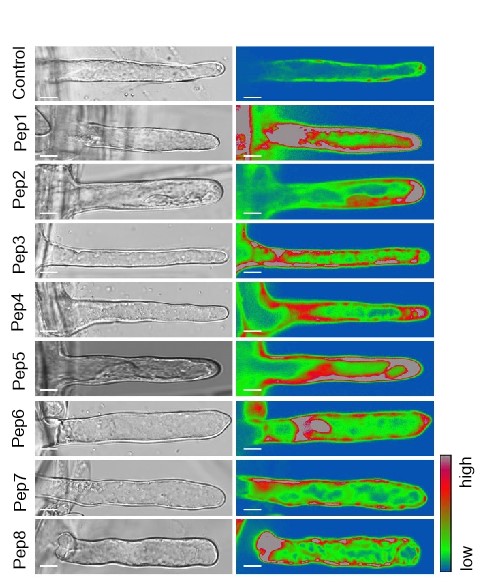


**Supplemental Figure 14. The root hair Ca^2+^ distribution.** The 6-day-old wild type WT plants expressing the genetically encoded intracellular Ca^2+^ indicator GCaMP6s were used. A pseudocolor scale bar for relative cytosolic Ca^2+^ level calibration is shown on the right. Bars = 10 μm.


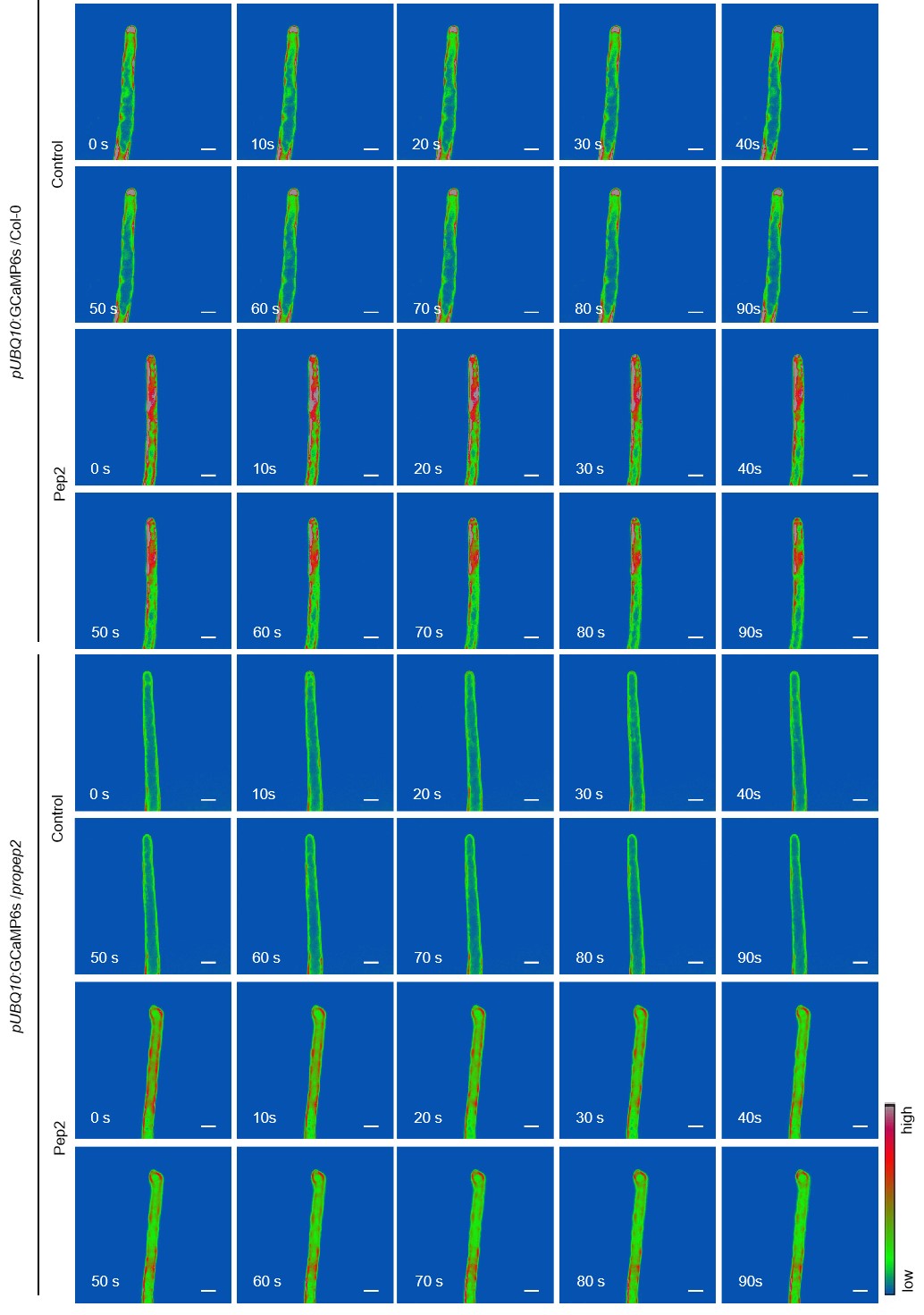


**Supplemental Figure 1**5. Imaging of Ca2+ fluorescence signals in the elongation root hairs of WT and propep2 plants. Six-day-old WT and propep2 plants expressing GCaMP6s were transplanted on half-strength Murashige and Skoog (MS) agar medium supplemented with or without 10 nm Pep2 for 6 h. The root hairs harboring GCaMP6s were monitored by a LSM-710 confocal microscope with a 20 × objective, The interval of data acquisition was 10 seconds, the Z-stack images were obtained with 1 μm steps and the scan speed was 6 s/scan. A pseudocolor scale bar for relative cytosolic Ca^2+^ level calibration is shown on the right. Bars = 10 μm.


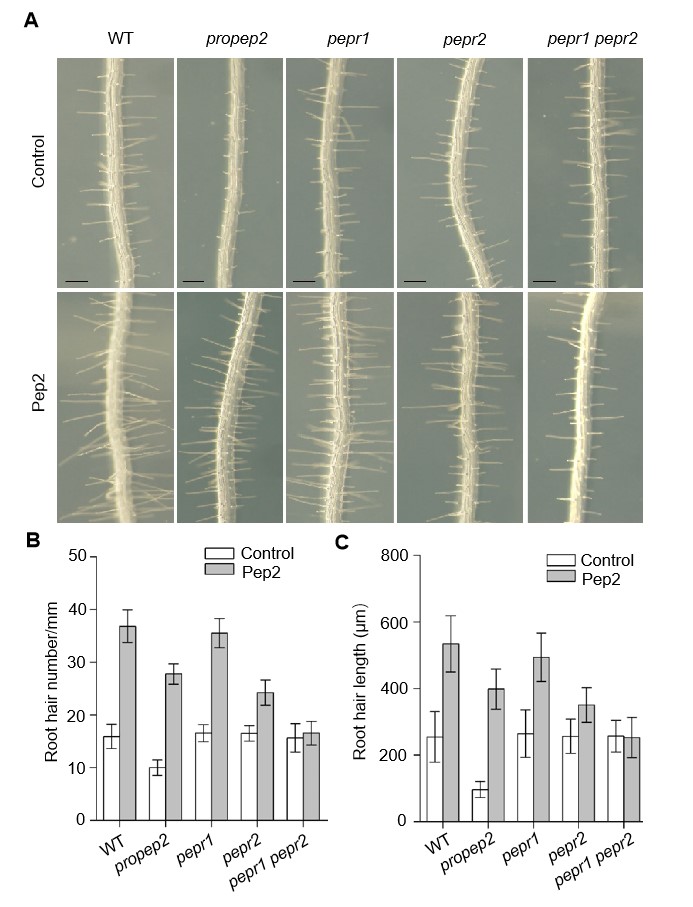


**Supplemental Figure 16. Pep2 regulates the root hairs development dependent on PEPRs.** (A) The growth phenotype of root hairs under Pep2 treatment. Four-day-old wild type (WT), *pepr1*, *pepr2* and *pepr1 pepr2* plants were transplanted on half-strength Murashige and Skoog (MS) agar medium supplemented with or without 10 nM Pep2 for 48 h. Bars = 200 μm. (B) and (C) Statistics of the root hair number (B) and root hair length (C) as in (A). Data are means ± SD (n = 15 roots per treatment).

**Supplemental Table 1. Primers used in this study.**

| Primer name | Primer sequences (5'-3') | Application |
| --- | --- | --- |
| PROPEP2-qRT-F | TAACCAGCCGGAGGAACAAG | qRT-PCR for gene expression |
| PROPEP2-qRT-R | TAACCAGCCGGAGGAACAAG |  |
| LB1 | TGGTTCACGTAGTGGGCCATCG | PCR and RT-PCR identification of homozygotes |
| PROPEP2-LP | ATGGAGAAATTAGATAAACGG |  |
| PROPEP2-RP | TTAATCCTCCTTATAAACTTGT |  |
| PROPEP1-RT-F | CGAAACAGCCGAAGGAGGAA |  |
| PROPEP1-RT-R | GGACGGCCTGAGCTAACTTT |  |
| PROPEP2-RT-F | ATGGAGAAATTAGATAAACGG |  |
| PROPEP2-RT-R | TTAATCCTCCTTATAAACTTGT |  |
| PROPEP3-RT-F | TGAAGTGTTCCGGTCTCGAA |  |
| PROPEP3-RT-R | TCCTTTTCCTGAACTTGGCGT |  |
| PROPEP4-RT-F | AACTTAGCTCTCACGAAGCA |  |
| PROPEP4-RT-R | AAAAATAAAGGACTCGTAGGAGTT |  |
| PROPEP5-RT-F | GAAGATGCAGCAAGAGAGAG |  |
| PROPEP5-RT-R | TAGTTACATGTCGTAGTCGTTAACTC |  |
| PROPEP6-RT-F | ATGGAAGTTAATGGAGAAGAAGA |  |
| PROPEP6-RT-R | ATTGTTTTGACCAGGTCG |  |
| PROPEP7-RT-F | ATGGAGGGAGAGGGAAGAAGAG |  |
| PROPEP7-RT-R | TACTTCTTGTGATATCGTCACCG |  |
| PROPEP8-RT-F | GGATGAAGAGAGACGAGACGAAG |  |
| PROPEP8-RT-R | TTGTGTAGCCTCCTCCGAA |  |
| GL2-F | ATCCGTGACAAACCTCGTCT | qRT-PCR analysis of gene  expression |
| GL2-R | ACGGTTCTTGCTCCTCTTGTT |  |
| CPC-F | ACGACGGAGACAGAGCAAAG |  |
| CPC-R | CGATCAACTCCCACCTGTCG |  |
| Actin2-F | CTGTTCTCTCCTTGTACGCCAGT |  |
| Actin2-R | CGGGTAATTCATAGTTCTTCTCGAT |  |
| PROPEP2-GD-F | CGGAATTCGCCGATATTGTCACTGTGGTT | Construction of PROPEP2 complementary lines |
| PROPEP2-GD-R | CGGGATCCGCCAAAGTGTGGTAGAAATTGTC |  |
| PROPEP1-GUS-F | ACGCGTCGACTTTACCTAGTAATCTAATACCATG | Tissue-specific expression analysis |
| PROPEP1-GUS-R | TCCCCCGGGTGAGATCTGATAAGACAGAGG |  |
| PROPEP2-GUS-F | GCTCTAGACTCTTGTGAATAGAGAAGAGAGAAC |  |
| PROPEP2-GUS-R | CGGGATCCTGAAATCCAATAGTTTGGTGAGTTATCG |  |
| PROPEP3-GUS-F | GCTCTAGACGCATTTAATATCCATCCATATCGAACG |  |
| PROPEP3-GUS-R | CGGGATCCCGTTGACTTCTTAATCTTTTTTTGGGAAAG |  |
| PROPEP4-GUS-F | GCTCTAGAAGCTTCCACAAGTCTTCGTTTCAAC |  |
| PROPEP4-GUS-R | CGGGATCCGTTTTTCTTCAATTCTGCTTCGTGAGAGC |  |
| PROPEP5-GUS-F | AACTGCAGCGTATAATTCCTGAAGATTGCAACTCC |  |
| PROPEP5-GUS-R | CGGGATCCCTTCGCTATCTTCTAAGTTCCTC |  |
| PROPEP6-GUS-F | GCTCTAGAGTCGTTTGTATAGGTCAGGTC |  |
| PROPEP6-GUS-R | CGGGATCCTCTTCTTCTTCTTCTTCTGCTCAATGC |  |
| PROPEP7-GUS-F | GCTCTAGATTGCAGCCGCCAATAAACCCTT |  |
| PROPEP7-GUS-R | CGGGATCCTCTTTCTTCTAACTACAAGAGATTGGGTC |  |
| PROPEP8-GUS-F | GCTCTAGAGGGTTCAAATCCCACTGTCAAC |  |
| PROPEP8-GUS-R | CGGGATCCTTAGATCCACTCTAATTGTCGGAAC |  |
| PROPEP1-PEZS-F | CGGAATTCGCATGGAGAAATCAGATAGACGAAGC | Subcellular localization assays in planta |
| PROPEP1-PEZS-R | CGGGATCCGCATTATGTTGGCCAGGACGGCCTG |  |
| PROPEP2-PEZS-F | CGGAATTCGCATGGAGAAATTAGATAAACGGAGGG |  |
| PROPEP2-PEZS-R | CGGGATCCGCATCCTCCTTATAAACTTGTATTGCCGC |  |
| PROPEP3-PEZS-F | CGGAATTCGCATGGAGAATCTCAGAAATGGAGAAG |  |
| PROPEP3-PEZS-R | CGGGATCCGCATTGTGTTTGCCTCCTTTTCCTG |  |
| PROPEP4-PEZS-F | CGGAATTCGCATGGAGAGAGGAGTTTCTTATTATC |  |
| PROPEP4-PEZS-R | CGGGATCCGCAAACGGCTTCTTGTTGGTGCCT |  |
| PROPEP5-PEZS-F | CGGAATTCGCATGCAGCAAGAGAGAGATCAC |  |
| PROPEP5-PEZS-R | CGGGATCCGCCATGTCGTAGTCGTTAACTCCAC |  |
| PROPEP6-PEZS-F | CGGAATTCGCATGGAAGTTAATGGAGAAGAAGAGAG |  |
| PROPEP6-PEZS-R | CGGGATCCGCTCAATTGTTTTGACCAGGTCGTCC |  |
| PROPEP7-PEZS-F | CGGAATTCGCATGGAGGGAGAGGGAAGAAGAG |  |
| PROPEP7-PEZS-R | CGGGATCCGCGTTGGTACCTCCACCCTTCC |  |
| PROPEP8-PEZS-F | CGGAATTCGCATGGATGAAGAGAGACGAGACG |  |
| PROPEP8-PEZS-R | CGGGATCCGCGTTGCGGCGGCCTGGCTTTC |  |
